# Accurate episomal HIV 2-LTR circles quantification using optimized DNA isolation and droplet digital PCR

**DOI:** 10.7448/IAS.17.4.19674

**Published:** 2014-11-02

**Authors:** Eva Malatinkova, Maja Kiselinova, Pawel Bonczkowski, Wim Trypsteen, Peter Messiaen, Jolien Vermeire, Bruno Verhasselt, Karen Vervisch, Linos Vandekerckhove, Ward De Spiegelaere

**Affiliations:** 1 Department of Internal Medicine, Ghent University and University Hospital Ghent, Ghent, Belgium; 2 Department of Infectious Diseases and Immunity, Jessa Hospital, Hasselt, Belgium; 3 Department of Clinical Chemistry and Microbiology, Ghent University and University Hospital Ghent, Ghent, Belgium

## Abstract

**Introduction:**

In HIV-infected patients on combination antiretroviral therapy (cART), the detection of episomal HIV 2-LTR circles is a potential marker for ongoing viral replication. Quantification of 2-LTR circles is based on quantitative PCR or more recently on digital PCR assessment, but is hampered due to its low abundance. Sample pre-PCR processing is a critical step for 2-LTR circles quantification, which has not yet been sufficiently evaluated in patient derived samples.

**Materials and Methods:**

We compared two sample processing procedures to more accurately quantify 2-LTR circles using droplet digital PCR (ddPCR). Episomal HIV 2-LTR circles were either isolated by genomic DNA isolation or by a modified plasmid DNA isolation, to separate the small episomal circular DNA from chromosomal DNA. This was performed in a dilution series of HIV-infected cells and HIV-1 infected patient derived samples (n=59). Samples for the plasmid DNA isolation method were spiked with an internal control plasmid.

**Results:**

Genomic DNA isolation enables robust 2-LTR circles quantification. However, in the lower ranges of detection, PCR inhibition caused by high genomic DNA load substantially limits the amount of sample input and this impacts sensitivity and accuracy. Moreover, total genomic DNA isolation resulted in a lower recovery of 2-LTR templates per isolate, further reducing its sensitivity. The modified plasmid DNA isolation with a spiked reference for normalization was more accurate in these low ranges compared to genomic DNA isolation. A linear correlation of both methods was observed in the dilution series (R2=0.974) and in the patient derived samples with 2-LTR numbers above 10 copies per million peripheral blood mononuclear cells (PBMCs), (R2=0.671). Furthermore, Bland–Altman analysis revealed an average agreement between the methods within the 27 samples in which 2-LTR circles were detectable with both methods (bias: 0.3875±1.2657 log_10_).

**Conclusions:**

2-LTR circles quantification in HIV-infected patients proved to be more accurate with a modified plasmid DNA isolation procedure compared to total genomic DNA isolation. This method enables the processing of more blood cells, thus enhancing quantification accuracy and sensitivity. An improved quantification of 2-LTR circles will contribute to the better understanding of ongoing replication in the HIV reservoir of patients on cART.

